# Tumid Lupus Erythematosus and Systemic Lupus Erythematosus: A Report on Their Rare Coexistence

**DOI:** 10.7759/cureus.7545

**Published:** 2020-04-05

**Authors:** Karan Jatwani, Karan Chugh, Oluwasegun S Osholowu, Shraddha Jatwani

**Affiliations:** 1 Internal Medicine, Mount Sinai St. Luke's Roosevelt Hospital Center, New York, USA; 2 Pulmonary Disease, Critical Care and Sleep Medicine, Einstein Medical Center, Philadelphia, USA; 3 Internal Medicine, Allina Health, Coon Rapids, USA; 4 Rheumatology, Einstein Medical Center, Philadelphia, USA

**Keywords:** tumid lupus, systemic lupus erythematosus, rash

## Abstract

Tumid lupus erythematosus (TLE) is a rare variant of cutaneous lupus erythematosus. Clinically, it lacks typical changes found in discoid lupus and antinuclear antibodies (ANA) levels are elevated in only 10% of the patients. Coexistent systemic lupus erythematosus (SLE) has been reported to be rare, and literature shows only a few case reports. We present a case of coexistent tumid lupus and SLE. We present a case of a 48-year-old Caucasian female who presented with chronic facial rash, photosensitivity, intermittent oral ulcers, joint pain with morning stiffness, and unintentional weight loss. Laboratory studies showed positive ANA at 1:640, elevated erythrocyte sedimentation rate, positive anticardiolipin immunoglobulin (Ig) G, anticardiolipin IgM, and anti-beta-2 glycoprotein IgM. Skin biopsy of the rash showed a superficial and deep dense lymphocytic infiltrate with mucin deposition, histopathology favoring tumid lupus. The patient was diagnosed with TLE with SLE and was started on hydroxychloroquine with improvement in her rash. Ultraviolet light and certain medications have been proven to play a role in the pathogenesis of tumid lupus. It usually responds to photoprotection, topical treatment, or oral antimalarial therapy.

## Introduction

Tumid lupus erythematosus, or lupus erythematosus tumidus, is a rare form of chronic cutaneous lupus erythematosus. Tumid lupus erythematosus was first reported by Erich Hoffman at a meeting in Berlin in 1909 [1]. Through the years, the classification of tumid lupus erythematosus has been controversial. Therefore, there are limited reported cases of the disease. Earlier ideas about tumid lupus erythematosus were that it is a unique category of intermittent cutaneous lupus erythematosus due to its recurrent course [1,2]. It was thought to be an extension of systemic lupus erythematosus or a form of chronic cutaneous lupus erythematosus such as discoid lupus erythematosus [3]. It was not until 2003 that Alexiades-Armenakas et al. proposed histopathologic and immunohistochemical criteria for the diagnosis of tumid lupus erythematosus, lending the strongest support yet to tumid lupus erythematosus as a distinct disease entity [4]. Clinically, tumid lupus erythematosus is known for its indolent and recurrent course, and its characteristic to heal without scarring. The histopathology of tumid lupus erythematosus comprises of a superficial and deep dense lymphocytic infiltrate in the perivascular and periadnexal regions, diffuse mucin deposition, and unaltered basement membrane. The majority of the lymphocytes in lesions of tumid lupus erythematosus are CD4 and less CD8 T cells. In the majority of reported cases, tumid lupus erythematosus is characterized by the low titer of antinuclear antibody (ANA) (≤1:160), with negative titers of other autoantibodies. We present a case of tumid lupus erythematosus, eventually developing systemic lupus erythematosus, with positive ANA.

## Case presentation

A 48-year-old Caucasian female with a past medical history of nephrolithiasis, gastroesophageal reflux disease, anxiety, and hyperlipidemia presented to a rheumatology clinic with facial rash, which had erupted two years prior to her clinic visit. The rash was noted to be pink, non-urticarial, erythematous, indurated, and without scarring or ulcerations. The patient reported that the rash was intermittent and photosensitive. The patient reported a history of hair loss, intermittent oral ulcers, photosensitivity, unintentional weight loss (10 pounds in one year), and arthralgias. She did not have any history of Raynaud’s phenomenon, history of thromboembolic events, hematuria, pleurisy, and pleural or pericardial effusions. She had no family history of systemic lupus erythematosus. 

**Figure 1:**
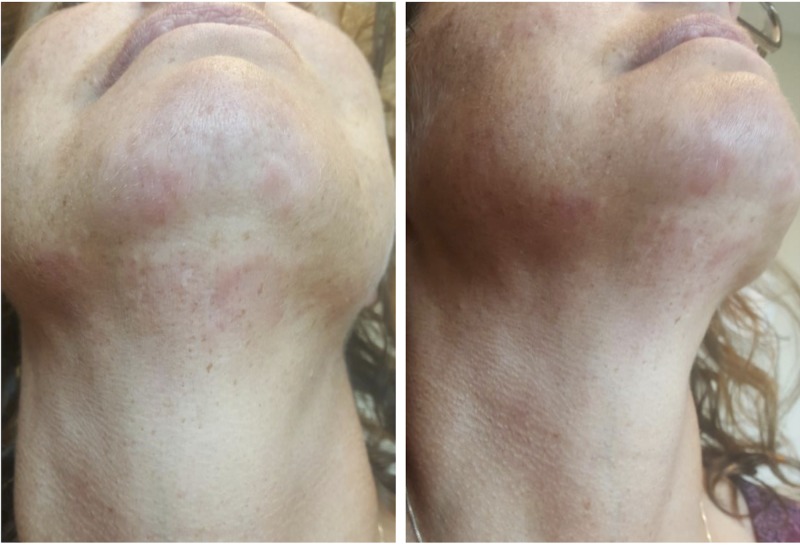
Facial rash in a patient with tumid lupus erythematosus over submandible and mandible, and mild over maxillae and nasal bridge.

The rash was prominent over the submandible and mandible, mild over the maxillae and nasal bridge, smooth, and without central clearing or scarring (Figure 1). She had applied daily topical 0.1% triamcinolone acetonide to the lesions over a few months, without resolution of the rash.

Workup for the rash showed elevated erythrocyte sedimentation rate 40 mm/hr, elevated ANA titers of 1:640 in a homogeneous pattern, anticardiolipin immunoglobulin (Ig) G of 27 GPL, anticardiolipin IgM of 94.4 MPL, and anti-beta-2 glycoprotein IgM of 47 SMU. Hepatitis C serology, rheumatoid factor, C-reactive protein, complement levels (C3 and C4), and antibodies to double-stranded DNA (dsDNA), Smith (Sm), cyclic citrullinated peptide, ribonucleoprotein, Sjogren’s antigen A, and Sjogren's antigen B were unremarkable. 

**Figure 2:**
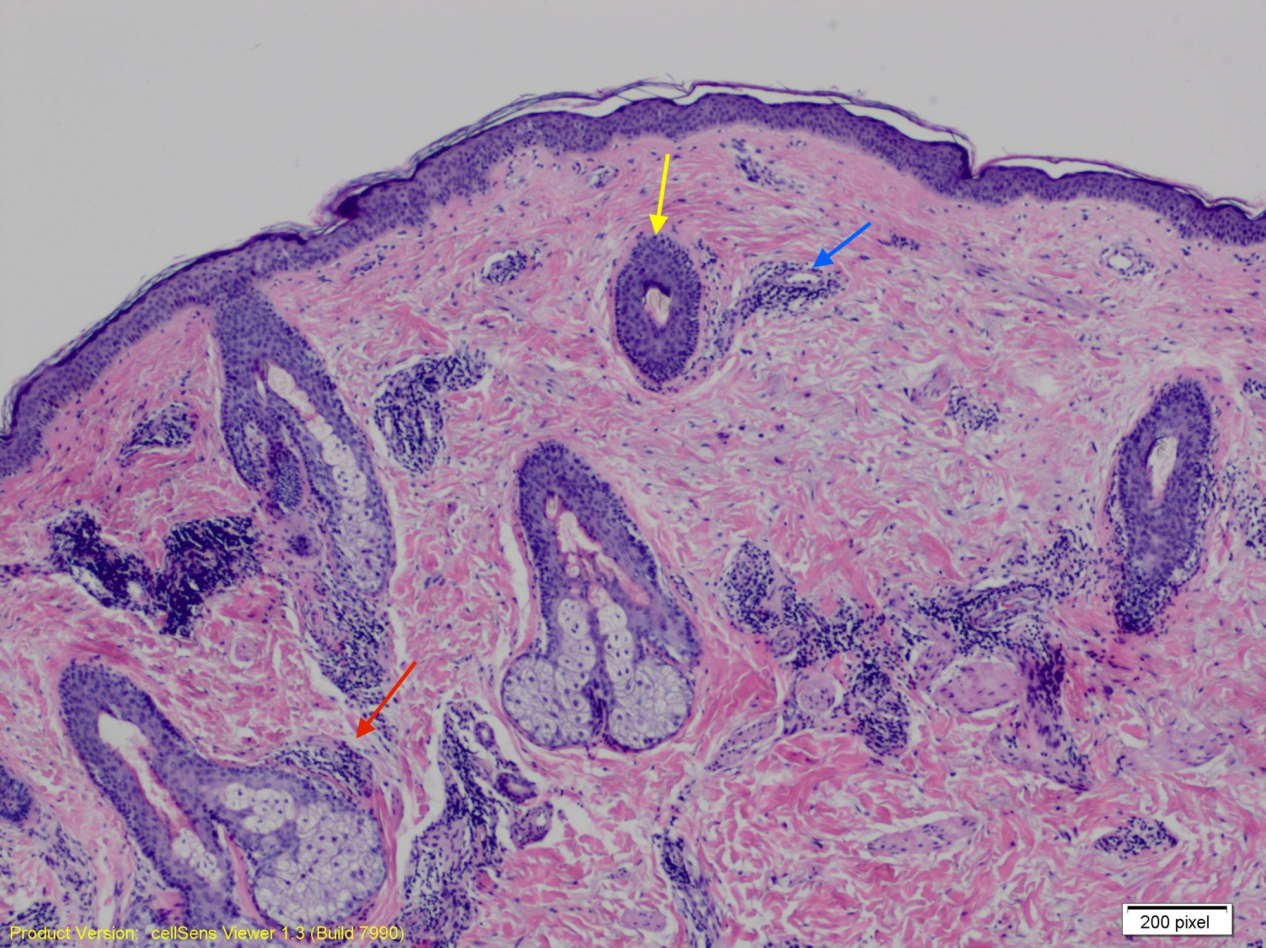
Skin biopsy showing perivascular (blue arrow) and periadnexal (hair follicle and sebaceous gland depicted by yellow and red arrow, respectively) lymphocytic infiltration within superficial and deep dermis. There is also diffuse mucin deposition throughout the dermal layer.

**Figure 3:**
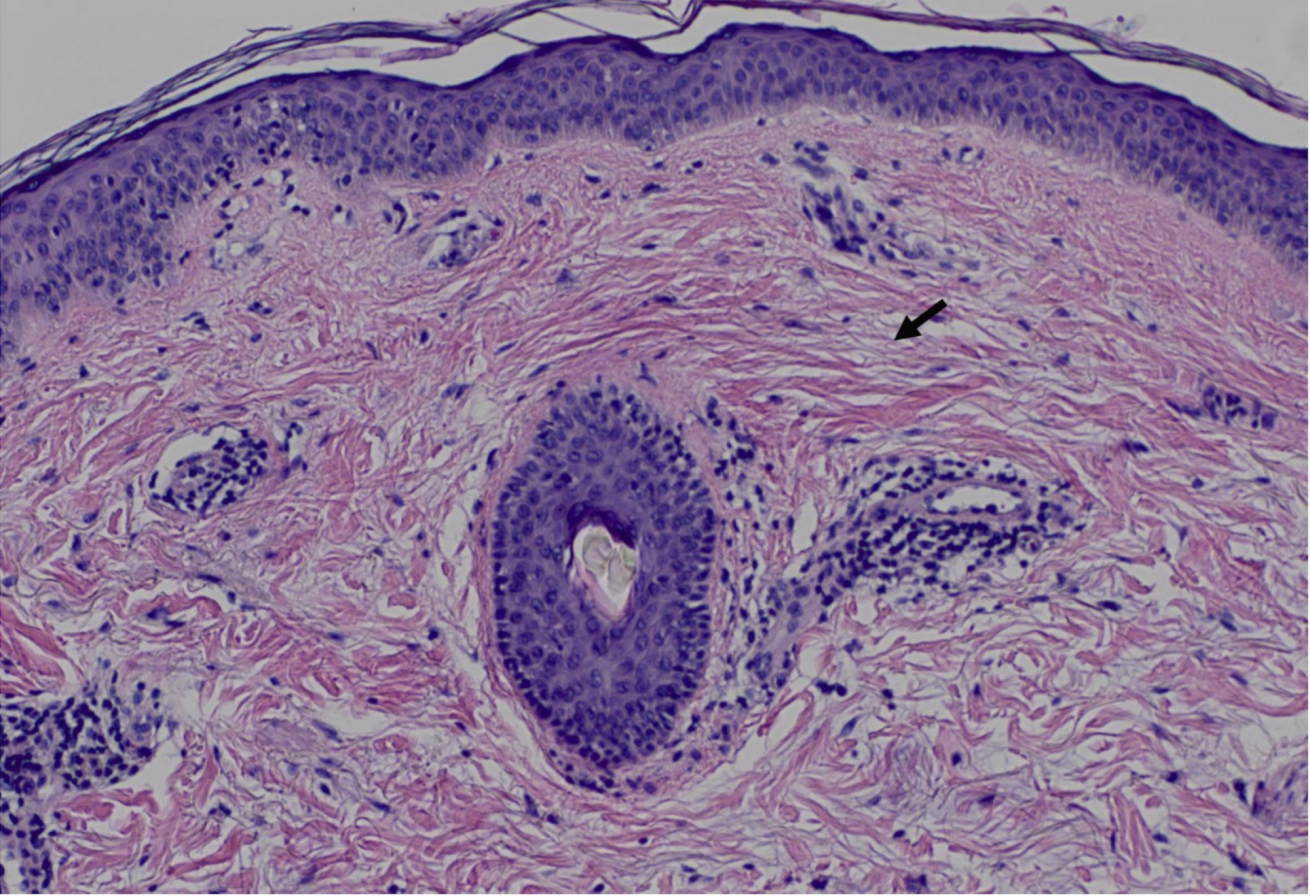
A close-up view of a superficial dermal layer from Figure 2. Diffuse mucin (black arrow) deposition throughout the dermal layer is better seen in this view.

A 0.4 x 0.4 x 0.2 cm punch biopsy of the patient’s right lateral chin showed a superficial and deep dermal perivascular and periadnexal lymphocytic infiltrate, and a normal dermal-epidermal junction. There was also a prominence of diffuse mucin deposition throughout the dermis (Figures 2, 3). 

The direct immunofluorescence of our sample was probed with fluorescein-labeled anti-human antibodies against IgG, IgA, IgM, C3, C5b-9, and fibrinogen. This returned positive for focal granules of IgM, C3, and C5b-9, which were more pronounced at the adnexa rather than the epidermal basement membrane zone.

The patient was diagnosed with tumid lupus erythematosus based on histopathology and direct immunofluorescence results, and clinical features. Other less likely but considered differentials were Jessner’s lymphocytic infiltrations, polymorphous light eruption, pseudolymphoma, and plaque mucinosis. And with her symptoms and signs of photosensitivity, oral ulcers, positive ANA, positive antiphospholipid antibodies, and abnormal serum level of IgG or IgM anticardiolipin antibodies, she was classified with systemic lupus erythematosus [5].

The patient was started on hydroxychloroquine sulfate 200 mg daily and continued on triamcinolone acetonide 0.1% topical cream daily with the resolution of the rash, and improvement in oral ulcers and arthralgias within three months.

## Discussion

Tumid lupus erythematosus is a rare variant of chronic cutaneous lupus erythematosus with only a few numbers of cases published to date. Over the years, the classification of tumid lupus erythematosus has been controversial. In 2003, Alexiades-Armenakas et al. proposed histopathologic and immunohistochemical criteria for the diagnosis of tumid lupus erythematosus, lending the strongest support yet to tumid lupus erythematosus as a distinct disease entity [4].

Tumid lupus erythematosus shares multiple characteristics with other chronic cutaneous lupus, which includes an indolent disease course. The lesions of tumid lupus erythematosus present as fixed, persistent cutaneous lesions that can last for months to years. There is a lack of gender preference, although studies have occasionally shown slight male predominance [5]. About 70% of tumid lupus erythematosus lesions are associated with photosensitivity, which is a common characteristic of cutaneous lesions of systemic lupus erythematosus. Clinical clues to suggest tumid lupus erythematosus from other chronic cutaneous lupus include the finding of lesions that are usually pink to violaceous, palpable, and non-scarring nodules. There is also a lack of surface changes, such as follicular plugs, atrophy, ulceration, or scaling, that are seen in other chronic cutaneous lupus erythematosus, such as in discoid lupus erythematosus or lupus profundus. Tumid lupus erythematosus is mainly a skin-limited disease and is the least likely out of the other types of chronic cutaneous lupus to occur concomitantly with systemic lupus erythematosus. 

Serologically, tumid lupus erythematosus is typically characterized by a low titer of ANA. In more than half of reported cases of tumid lupus erythematosus, ANA titers were low (≤1:160) with other autoantibody panels being negative [4]. The ANA titers in these cases are inconsistent, with only a few cases documenting high titers (≥1:160) of ANA, at the time of diagnosis of tumid lupus erythematosus [6,7]. In our case, the patient’s ANA titer was 1:640. This finding might be explainable with the presence of symptoms of systemic lupus, with cardiolipin and beta-2 glycoprotein antibodies. There have been only a few reported cases in which tumid lupus erythematosus was diagnosed in patients either with a history of or with concomitant systemic lupus erythematosus or discoid lupus erythematosus [4-8]. Even though the occurrence of tumid lupus erythematosus and systemic lupus erythematosus is rare, it would increase morbidity and mortality. Hence, we suggest that patients diagnosed with tumid lupus erythematosus should be evaluated for systemic lupus erythematosus. A high ANA titer should prompt further workup to include anti-dsDNA, anti-Sm, complete blood count with differential, creatinine, erythrocyte sedimentation rate, urinalysis, 24-hour urine protein, 24-hour urine creatinine, complement levels (C3 and C4), and antiphospholipid antibody testing.

Histopathologically, our sample was consistent with the classic findings of tumid lupus erythematosus, which includes a superficial and deep dense lymphocytic infiltrate in the perivascular and periadnexal regions, and diffuse mucin deposition. The basement membrane was unaffected. The direct immunofluorescence of our sample was probed with fluorescein-labeled antihuman antibodies against IgG, IgA, IgM, C3, C5b-9, and fibrinogen, and was positive for focal granules of IgM, C3, and C5b-9, which were more pronounced at the adnexa rather than the epidermal basement membrane zone. In previous studies, direct immunofluorescence has also been used to show a CD4 predominance over CD8 T cells in the lymphocytic infiltrate of tumid lupus erythematosus [4,9].

The rash of tumid lupus erythematosus tends to follow a chronic, recurrent, and photosensitive pattern. Tumid lupus erythematosus has a better prognosis than most cutaneous lupus erythematosus. Spontaneous resolution of the rash is possible, although tumid lupus erythematosus is known to react well to a combination of photoprotection, topical corticosteroids and oral antimalarials as the first-line treatment. Second-line treatments include methotrexate and mycophenolate, while third-line treatments are thalidomide and lenalidomide. The rash of tumid lupus erythematosus is known to heal without skin disfiguration, scarring, and dyspigmentation. Complete resolution of the rash might take up to three months with treatment. 

## Conclusions

Tumid lupus erythematosus has a better prognosis than most cutaneous lupus erythematosus. It can be one of the presenting features for underlying systemic lupus erythematosus, and patients diagnosed with tumid lupus erythematosus should be evaluated for systemic lupus erythematosus.
